# Fehling Test for Sugar

**Published:** 1907-11

**Authors:** 


### FEHLING TEST FOR SUGAR.

McLean enumerates and explains the action of the different substances in the urine which interfere with the Fehling test or give an ambiguous reaction. In regard to the general significance of these ambiguous reactions he says that every urine contains a certain amount of sugar which is prevented by the creatinin present from giving any reaction with the Fehling test. Concentrated to half the amount, and containing consequently double the amount of sugar, such a specimen would give a doubtful Fehling reaction. He advises in doubtful cases, where the specific gravity is high, that it be reduced by the addition of water to about 1015.

In urines containing a slightly higher percentage of sugar than normal the important point, of course, is to determine how much urine is passed per diem, and so obtain a clue as to the total sugar passed. An ambiguous reaction with Fehling's test in the urine of an individual passing less than 100 c.c. per day would obviously have a different significance to that of a urine giving a somewhat similar reaction, but of which the daily average amount was nearly 200 c.c., or even over this. In a case giving a modified reaction with Fehling's test after dilution it is always advisable to postpone a report until a future examination can be made, for it seems certain that the presence of small amounts of sugar in urine in quantities above the normal is often caused by factors which have very little direct bearing on the probability of life. The amount of exercise taken, the quantity of carbohydrate eaten, working or sitting in badly-ventilated rooms or offices, temporary feverish or mentally-disturbed conditions, alcohol, etc., all tend to cause a slight increase in the amount of sugar, excreted in the urine of many apparently healthy individuals.

Again observation proves that sugar may be occasionally present in the urine of healthy people without any apparent cause, and that the power of assimilation of carbohydrates may vary within fairly wide limits, not only in different individuals, but in the same individual. The important point to consider is whether, with ordinary diet, the urine always contains a small excess of sugar. If it does so, the case is suspicious. If the sugar is of but temporary occurrence it can be said that in the majority of cases its presence is of no importance. This temporary increase of sugar is often observed in cases of mental disturbance.

The fact that every urine contains a certain amount of sugar, that the amount normally present varies with different individuals and probably for the same individual, and that the test is at best but a relative one, indicates that the occurrence of an ambiguous reaction in a concentrated urine is generally of no clinical importance, and though largely due to carbodydrates, does not usually indicate an abnormal condition. In urines of low specific gravity these ambiguous reactions do generally indicate a slight excess of sugar, but its probable bearing and importance can only be ascertained by watching the case, and by repeated examination of the urine at short intervals. In many cases it will be found to disappear and give no further trouble; in a few it may show the starting point of a true diabetes mellitus, and in such cases early detection of the true condition is exceedingly important.—British Med. Jour.

